# Validation of Noninvasive Remote Dielectric Sensing System to Quantify Lung Fluid Levels

**DOI:** 10.3390/jcm11010164

**Published:** 2021-12-29

**Authors:** Teruhiko Imamura, Wataru Gonoi, Masakazu Hori, Yohei Ueno, Nikhil Narang, Hiroshi Onoda, Shuhei Tanaka, Makiko Nakamura, Naoya Kataoka, Ryuichi Ushijima, Mitsuo Sobajima, Nobuyuki Fukuda, Hiroshi Ueno, Koichiro Kinugawa

**Affiliations:** 1 Second Department of Internal Medicine, University of Toyama, Toyama 9300194, Japan; masahori6059@yahoo.co.jp (M.H.); fef6ge@gmail.com (Y.U.); ohiro0203@gmail.com (H.O.); stanaka@med.u-toyama.ac.jp (S.T.); nakamuramk1979@gmail.com (M.N.); nkataoka@icloud.com (N.K.); ryuryu0702@gmail.com (R.U.); soba1126@yahoo.co.jp (M.S.); nfukuda@med.u-toyama.ac.jp (N.F.); hueno@med.u-toyama.ac.jp (H.U.); kinugawa-tky@umin.ac.jp (K.K.); 2 Department of Radiology, Graduate School of Medicine, University of Tokyo, Tokyo 1138654, Japan; gonoiw@gmail.com; 3 Advocate Christ Medical Center, Oak Lawn, IL 60453, USA; nikhil.narang@gmail.com

**Keywords:** congestion, heart failure, hemodynamics, CT densitometry

## Abstract

Background: The accuracy of the remote dielectric sensing (ReDS^TM^) system, which is a noninvasive electromagnetic-based technology to quantify lung fluid levels, particularly among those with small body size, remains uncertain. Methods: Hospitalized patients with and without heart failure underwent assessment of lung fluid levels with ReDS and successive chest computed tomography imaging. We performed a correlation analysis of the ReDS measurement, representing lung fluid levels, and computed tomography-derived high attenuation area percentage, which also provides a spatial quantification of lung fluid level. Results: A total of 46 patients (median 76 years old, 28 men), including 28 patients with heart failure, were included. The median ReDS value was 28% (interquartile: 23%, 33%), and the median percentage of high attenuation area was 21.6% (14.4%, 28.5%). ReDS values and percentage of high attenuation area were moderately correlated (r = 0.65, *p* < 0.001), irrespective of the existence of heart failure. ReDS value independently predicted the percentage of high attenuation area seen on computed tomography (*p* < 0.001). Conclusions: The ReDS system may be a promising, noninvasive tool to quantify fluid lung levels, as validated by comparison with chest computed tomography imaging. Further studies are warranted to validate the utility and applicability of this technology to a variety of clinical scenarios.

## 1. Introduction

The significant morbidity and mortality benefit of neurohormonal antagonists in patients with chronic heart failure has been shown in large-scale randomized control trials. Comprehensive four-tier medical therapy for chronic heart failure with reduced ejection fraction including angiotensin-neprilysin inhibitors, beta-blockers, mineralocorticoid receptor antagonists, and sodium-glucose cotransporter 2 inhibitors can offer dramatic additional clinical risk reduction compared to angiotensin converting enzyme inhibitors and beta-blockers alone [1]. Promising data have also recently emerged demonstrating the benefit of both angiotensin-neprilysin inhibitors and sodium-glucose cotransporter 2 inhibitors in patients with heart failure and preserved ejection fraction [2,3].

Nevertheless, the management of residual pulmonary congestion is an important treatment goal given its impact on patient quality of life [4]. However, given the lack of a gold-standard to accurately assess lung fluid levels, precise management of pulmonary congestion remains a clinical challenge. Chest computed tomography (CT) can quantify the presence of pleural and interstitial lung fluid, but is cumbersome for monitoring, poses a risk of radiation, and comprises a highly complex methodology.

Recently, a remote dielectric sensing system (ReDS^TM^, Sensible Medical Innovations Ltd., Netanya, Israel), a noninvasive electromagnetic-based technology to quantify lung fluid volumes, was introduced for clinical use [5]. A preliminary analysis observed a significant correlation between ReDS values and lung fluid amount measured by high-resolution CT [6]. Additional contemporary analyses have also shown the benefit of ReDS measurements in the guidance of heart failure management [7,8]. However, these studies were conducted in Western countries and did not include subjects with smaller body sizes. In this study, we investigated the correlation between ReDS values and lung fluid levels, measured by chest CT imaging to validate ReDS quantification of pulmonary congestion in a broad range of body sizes.

## 2. Methods

### 2.1. Participant Selection

In this prospective study, hospitalized patients with and without heart failure received ReDS measurements following clinical stabilization. Chest CT imaging was performed on the same day following the ReDS measurements. Patients who were unable to have ReDS measurements taken, including those with intrathoracic devices such as permanent pacemakers, or pulmonary lesions including lung malignancies and pneumonia, were excluded. Informed consent was obtained from all participants beforehand. The institutional ethical review board approved the study protocol (MTK2020007).

### 2.2. ReDS System

**Figure 1:**
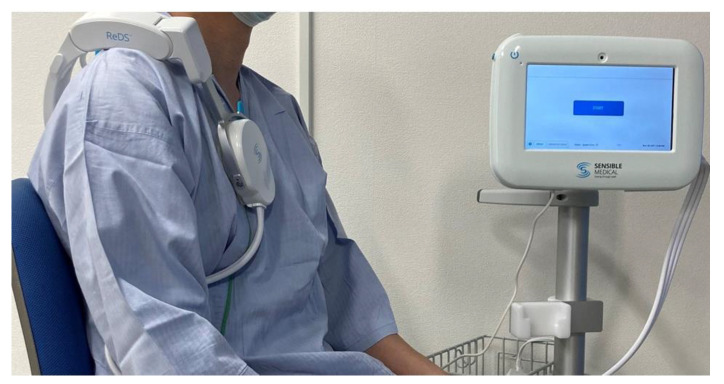
A ReDS system consisting of a monitor and a sensor unit.

The ReDS system estimates lung fluid levels and degree of pulmonary congestion under natural breathing conditions [5]. ReDS employs low-power electromagnetic signals emitted between two sensors embedded on a wearable device (Figure 1). The analyzed signals reflect the dielectric properties of the lung portion between the two sensors. The dielectric coefficients of a material are represented by a frequency-dependent number describing its interaction with electromagnetic energy, including the degrees of reflection, absorption, and transmission of the energy. Given that water has a high dielectric coefficient and air has a low one, the dielectric coefficient of tissue is determined predominantly by its fluid content. The normal range for the ReDS value, as proposed by the product’s manufacturer, is between 20% and 35%. 

### 2.3. CT Image Acquisition

All patients underwent CT scan examinations in the supine position at full inspiration without intravenous contrast. CT images were acquired using a dual-source CT unit (SOMATOM Force scanner, Siemens Healthineers, Erlangen, Germany). The scanning parameters were as follows: tube voltage, 120 kVp; tube current, controlled by automated tube current modulation; matrix size, 512 × 512; pixel spacing, 0.625 mm × 0.625 mm. CT images of 5-mm slice thickness were reconstructed using lung reconstruction kernels.

#### Automated Volume Analysis of the CT Images

A commercially available CT imaging analysis workstation, the Synapse VINCENT Ver 6.4.0003 (Fujifilm Medical Systems, Tokyo, Japan), was used for volumetric analysis of hyperattenuated lungs [9,10].

First, an expert diagnostic radiologist who was blinded to the clinical data including ReDS values evaluated the imaging to rule out the presence of lung lesions other than pulmonary congestion and mild emphysema, along with those with pneumonias and neoplasms, who were excluded.

**Figure 2:**
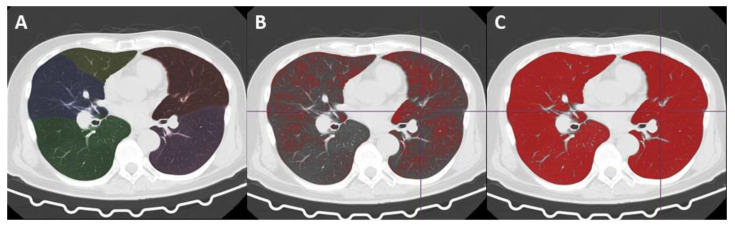
Automated volume analysis of the CT images. Chest computed tomography (CT) image of a 73-year-old man with suspected pulmonary hypertension. Automatically extracted and segmented lungs and lobes are color-coded (**A**). Red-painted areas indicate lung parenchyma with and under −950 HU (**B**) and −750 HU (**C**), which amounted to 21.7% and 91.6% of the whole lung (whole volume, 6456.6 mL; average density, −885.1 HU), respectively. Therefore, this patient’s %high attenuation area (from −749 HU to 0 HU) was calculated as 8.4% of the whole lung.

Second, using a lung analysis tool for automatic whole lung extraction from the chest, CT images were taken excluding the thoracic wall, mediastinum, large vessels, pleural effusion, and airways (from the trachea to tertiary bronchi) (Figure 2A–C).

Third, a threshold-based volumetric CT analysis was performed to estimate the volume of hyperattenuated lung, which indicates pulmonary congestion, and its ratio to whole lung volume.

Although there is no established CT value threshold for normally attenuated lung and lungs with pulmonary congestion, based on several past threshold-based analyses for various lung diseases, we set the lower and upper limit CT value for a normal lung at −950 HU and different CT values to within the range from −800 to −500, respectively [11,12,13,14,15].

A past study reported that early pulmonary congestion and edema due to left-sided heart disease caused a rise in CT values, prominently in the range between −750 and −650 HU, suggesting that −750 HU is a reasonable candidate threshold for distinguishing normal lung and pulmonary congestion [16].

Finally, we defined the threshold for the present study as follows: the whole lung, from −1000 HU to 0 HU; low attenuation area (emphysematous lung), −1000 HU to 950 HU; normal attenuation area (normal lung), from −949 HU to −750 HU; high attenuation area (edematous lung), from −749 HU to 0 HU.

In the process of lung extraction and threshold-based analysis, the volumes and mean CT values were available for each lung portion. The radiologist checked all the processed images and measured values, and confirmed that the analyses had been performed correctly.

### 2.4. Statistical Methods

Continuous variables are presented as medians and interquartiles. Categorical variables are presented as numbers and percentages. The correlation between ReDS values and %high attenuation area (volumetric ratio of high attenuation area to the whole lung) was assessed using Pearson’s correlation. Linear regression analyses were conducted to investigate the impact of baseline characteristics, including ReDS values, on the %high attenuation area. All analyses were performed in SPSS Statistics 23.0 software (IBM Corp, Armonk, NY, USA), and two-tailed *p* values less than 0.05 were assumed significant.

## 3. Results

### 3.1. Baseline Characteristics

**Figure 3:**
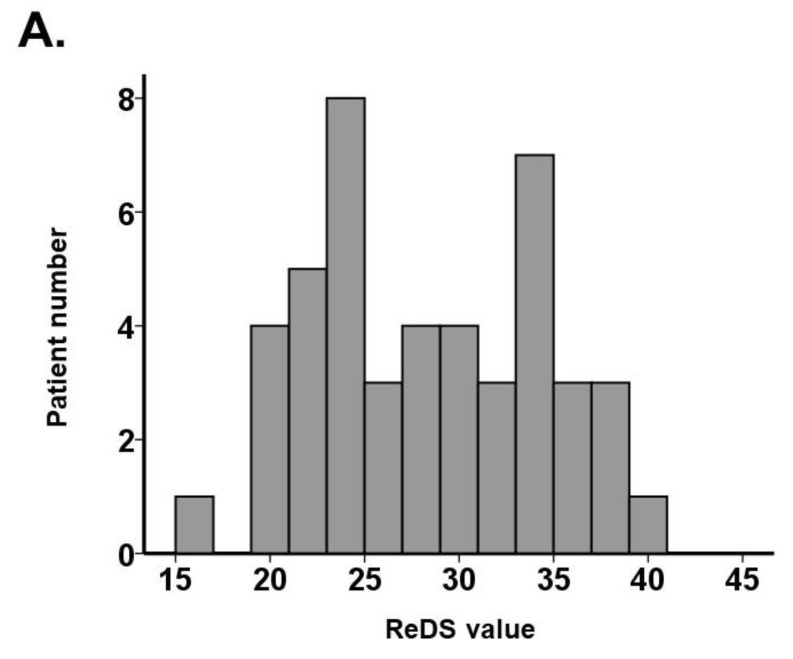

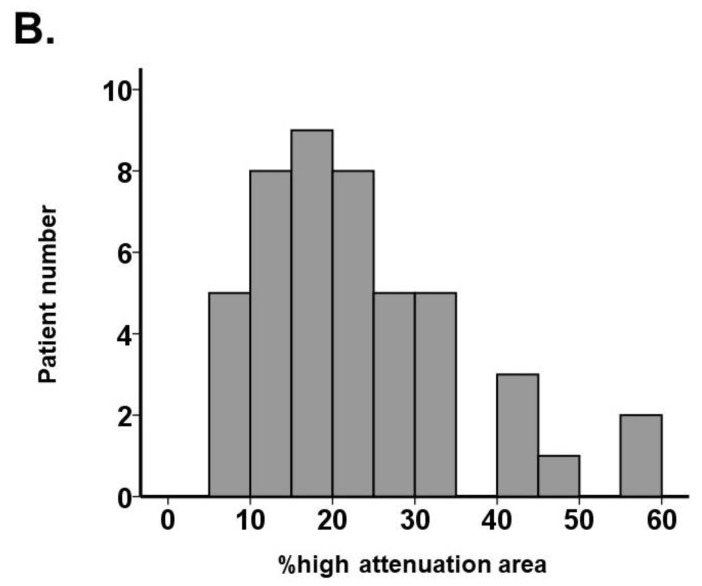
Distribution of ReDS value (**A**) and %high attenuation area (**B**).

A total of 46 hospitalized patients were included (Table 1). The median age was 76 (73, 84) years old, and 28 (61%) subjects were men. The median body mass index was 21.6 (19.7, 26.0). The distribution of ReDS values and %high attenuation area are shown in Figure 3A,B. The median ReDS value was 28% (23%, 33%), and the median %high attenuation area was 21.6% (14.4%, 28.5%).

Within the overall cohort, 28 patients had heart failure. Other reasons for admission were ischemic heart disease, pulmonary hypertension, and acute kidney injury. Heart failure patients had a worse ventricular function and higher plasma B-type natriuretic peptide (Table 1). The ReDS value and %high attenuation area tended to be higher in the heart failure group (Table 1).

### 3.2. ReDS Value and %High Attenuation Area

**Figure 4:**
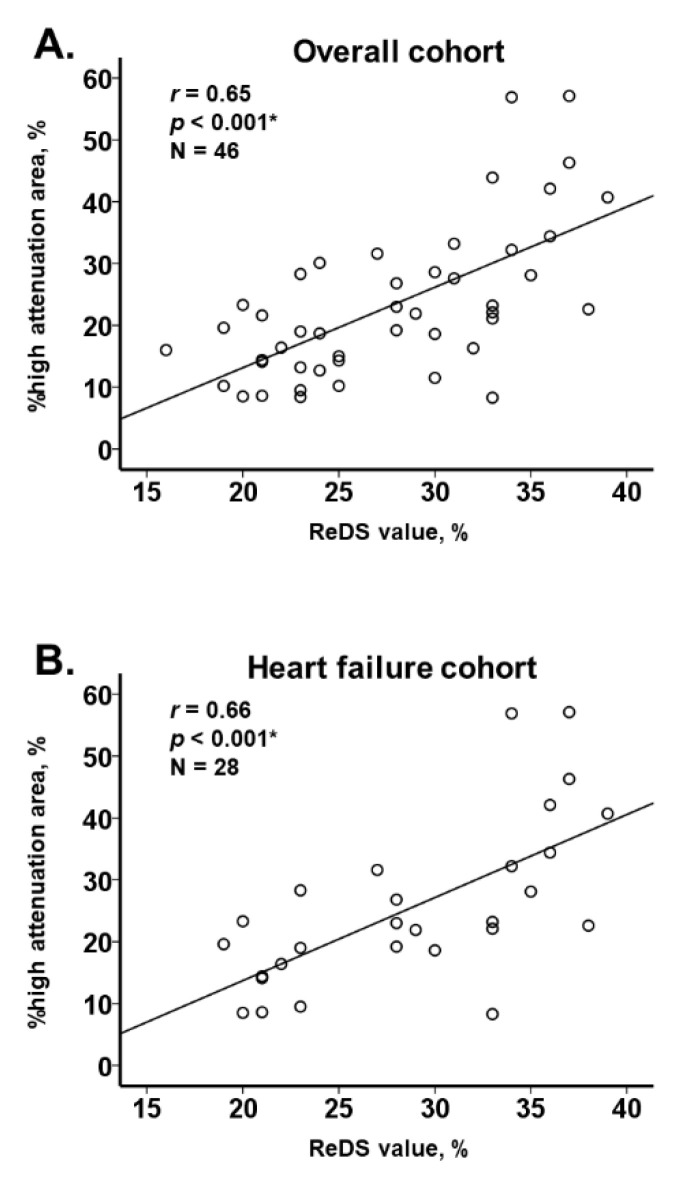

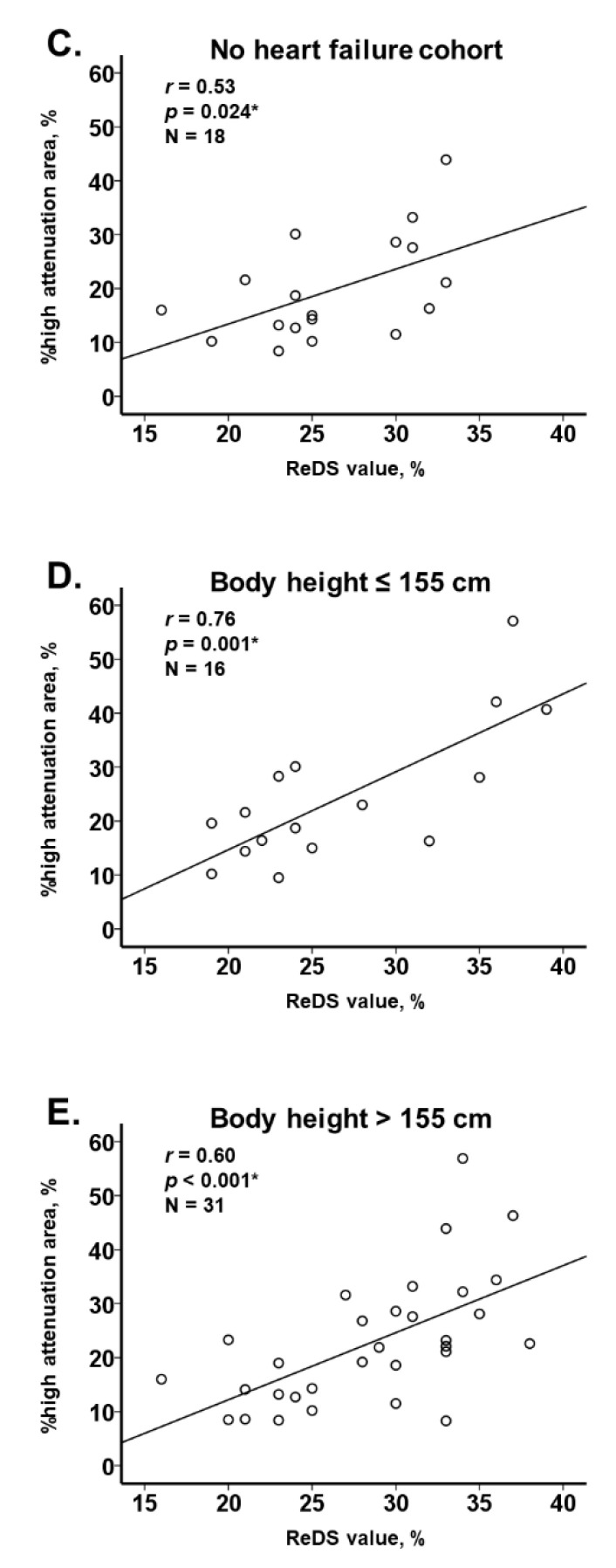
Correlation between ReDS value and %high attenuation area among all cohort (**A**), those with and without heart failure (**B**,**C**), and those with and without body weight ≤155 cm (**D**,**E**). * *p* < 0.05 by Pearson’s correlation coefficient.

**Figure 5:**
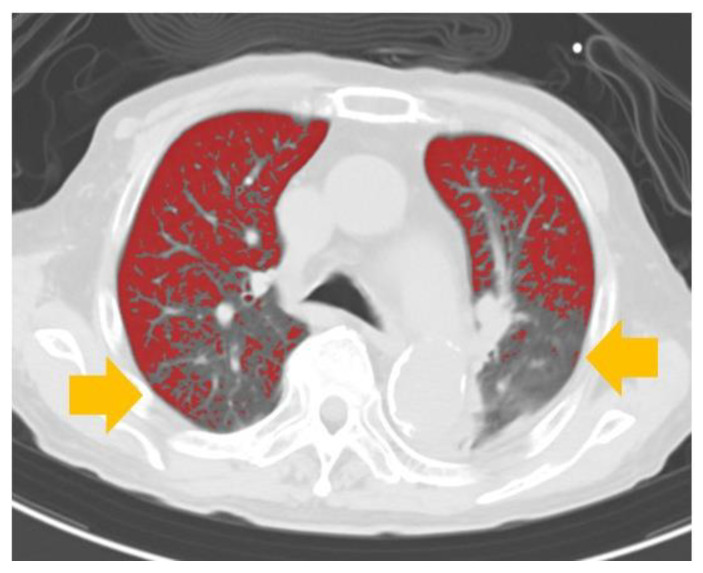
A representative case of pulmonary congestion. Chest CT image of a 75-year-old man with aortic stenosis and heart failure showing 34% ReDS and 56.9% high attenuation area. Yellow arrows in the bilateral lungs indicate hyperattenuated areas due to pulmonary congestion.

ReDS values moderately correlated with %high attenuation area in the overall cohort (r = 0.65, *p* < 0.001; Figure 4A), heart failure cohort (r = 0.66, *p* < 0.001; Figure 4B), and nonheart failure cohort (r = 0.53, *p* = 0.024; Figure 4C). A moderate correlation was observed among those with body height ≤155 cm (r = 0.76, *p* = 0.001, N = 16; Figure 4D) and those with body height >155 cm (r = 0.60, *p* < 0.001; N = 31; Figure 4E). A representative CT image with elevated ReDS value and %high attenuation area (34% and 56.9%, respectively) is displayed in Figure 5. Yellow arrows indicate bilateral pulmonary congestion.

The ReDS value was an independent predictor of %high attenuation area after adjusting for two potential confounders (body mass index and plasmas B-type natriuretic peptide) (*p* < 0.001; Table 2).

## 4. Discussion

In this study, we observed a moderate positive correlation between the ReDS value and %high attenuation area, both of which estimate lung fluid amount, irrespective of the presence of heart failure and body size.

### 4.1. Conventional Methodologies to Assess Pulmonary Congestion

Early and accurate methodologies to assess pulmonary congestion for patients prior to developing dyspnea are needed to curb the potential for rehospitalization due to heart failure, which is independently associated with a worse clinical prognosis.

Physical examination and chest X-ray are conventional and practical tools to assess for pulmonary congestion. However, they require expert technique and are often inaccurate, with intracardiac pressures often being underestimated in patients with chronic heart failure [17]. Plasma B-type natriuretic peptide is used as a surrogate of clinical risk in patients with chronic heart failure. The value reflects ventricular wall stress and distension, and does not always accompany pulmonary congestion. Various modifiers, including the presence of obesity, baseline renal function, age, and sex, may affect the interpretation of the absolute value [18]. Echocardiography may offer indirect surrogates to estimate filling pressures but does not universally quantify the degree of pulmonary congestion.

### 4.2. ReDS versus Invasive Hemodynamic Measurement

Right heart catheterization is the most accurate instantaneous method to quantify the intracardiac hemodynamics of heart failure [19]. However, patients with low cardiac output may, at times, have no pulmonary congestion despite incremental intracardiac pressures. Furthermore, given its invasiveness, right heart catheterization may not be feasible under certain circumstances. An intermediary is needed, and given its high sensitivity, the ReDS system may be a useful screening tool in patients with suspected pulmonary congestion who would require further intensive assessment including right heart catheterization [20].

### 4.3. ReDS System and Chest CT

We demonstrated a moderate correlation between the ReDS value and %high attenuation area calculated by chest CT, irrespective of body size. Patients with smaller body sizes, particularly those with a height ≤155 cm, were not included in previous studies [6,7,8].

CTs require radiation exposure and expert interpretation. The ReDS system has the advantages of being noninvasive, simple to use and providing data which is easy to interpret, all of which allow repeated assessments of pulmonary congestion on follow-up, screening, and discharge.

In contrast, chest CT can visually assess intrathoracic abnormalities in detail, including pneumonic processes and pleural effusions. Chest CT can be applied irrespective of thoracic anatomical abnormalities. The ReDS system is based on the hypothesis that pulmonary congestion is uniform among the whole lung, and focuses specifically on the right lung field [5]. The ReDS system might be inappropriate to assess heterogeneously distributed pulmonary congestion.

As a whole, %high attenuation areas were relatively lower than ReDS value. We might have to consider the difference of breathing situation between the two modalities. Patients held their breath at full inspiration during the CT imaging scan. In contrast, ReDS values are measured during natural breathing. The lung is filled with air at full inspiration, and the %high attenuation area might be relatively underestimated compared to that obtained during natural breathing.

### 4.4. Study Limitations

The present study was a proof-of-concept, using the ReDS system for the first time before commercial marketing in Japan. We enrolled only a small cohort. Patients received ReDS measurements after hemodynamic stabilization. The applicability of our findings to those with unstable hemodynamics with significant pulmonary congestion remains uncertain. Further studies are warranted to validate the applicability of this technology to various other clinical situations. At present, we recommend the use of ReDS during screening and follow-up. In critical situations, we recommend multimodalities instead of ReDS alone. We performed linear regression analyses considering that certain clinical conditions may have affected the ReDS values. However, there may have been other, uninvestigated confounders.

## 5. Conclusions

The ReDS system is a promising, noninvasive and easy-to-measure tool to quantify fluid lung fluid levels.

## Figures and Tables

**Table 1:** Baseline characteristics.

	Total (N = 46)	Heart Failure (N = 28)	Nonheart Failure (N = 18)	*p* Value
Demographics				
Age, years	76 (73, 84)	75 (72, 81)	79 (74, 86)	0.86
Men	28 (61%)	16 (57%)	12 (67%)	0.37
Body height, cm	159 (150, 167)	161 (151, 168)	157 (149, 166)	0.45
Body mass index, kg/m^2^	21.6 (19.7, 26.0)	20.8 (19.4, 23.9)	23.8 (21.5, 26.7)	0.087
Comorbidity				
Hypertension	33 (72%)	23 (82%)	10 (56%)	0.054
Dyslipidemia	26 (57%)	17 (61%)	9 (50%)	0.34
Diabetes mellitus	19 (41%)	12 (43%)	7 (39%)	0.52
Chronic kidney disease	24 (52%)	14 (50%)	10 (56%)	0.47
History of stroke	8 (17%)	5 (18%)	3 (17%)	0.92
Coronary heart disease	7 (15%)	2 (7%)	5 (28%)	0.057
Atrial fibrillation	9 (20%)	7 (25%)	2 (11%)	0.25
Echocardiography				
Left atrial diameter, mm	41 (36, 46)	40 (33, 46)	43 (41, 46)	0.29
Left ventricular end-diastolic diameter, mm	48 (45, 55)	51 (46, 56)	46 (43, 49)	0.010 *
Left ventricular ejection fraction, %	55 (47, 67)	51 (38, 59)	67 (58, 75)	<0.001 *
Mild or greater aortic regurgitation	14 (30%)	11 (39%)	3 (17%)	0.095
Mild or greater mitral regurgitation	23 (50%)	17 (61%)	6 (33%)	0.065
Mild or greater tricuspid regurgitation	20 (43%)	13 (46%)	7 (39%)	0.42
Laboratory data				
Hemoglobin, g/dL	11.9 (10.3, 13.4)	11.9 (10.8, 13.1)	11.9 (10.0, 14.4)	0.41
Serum albumin, g/dL	3.6 (3.1, 3.9)	3.6 (3.3, 4.0)	3.6 (2.8, 3.9)	0.63
Serum sodium, mEq/L	139 (137, 142)	140 (138, 142)	139 (137, 141)	0.42
eGFR, mL/min/1.73 m^2^	47.1 (31.1, 63.2)	47.5 (29.3, 63.2)	45.1 (31.1, 63.0)	0.61
Plasma B-type natriuretic peptide, pg/mL	207 (53, 501)	398 (179, 834)	42 (16, 152)	<0.001 *
Medication				
Beta-blocker	19 (41%)	14 (50%)	5 (28%)	0.12
Renin-angiotensin system inhibitor	24 (52%)	18 (64%)	6 (33%)	0.040 *
Mineralocorticoid receptor antagonist	10 (22%)	6 (21%)	4 (22%)	0.61
Loop diuretics	14 (32%)	8 (29%)	6 (33%)	0.49
ReDS value, %	28 (23, 33)	28 (23, 34)	25 (24, 31)	0.21
%high attenuation area, %	21.6 (14.4, 28.5)	22.6 (17.5, 30.0)	17.5 (12.1, 28.1)	0.10

eGFR, estimated glomerular filtration ratio; ReDS, remote dielectric sensing. * *p* < 0.05. Continuous variables are presented as median and interquartile and compared between the two groups using Mann-Whitney U test. Categorical variables are presented as numbers and percentages and compared between the two groups using Fischer’s exact test.

**Table 2:** Association between %high attenuation area and clinical variables including ReDS value.

	Univariate Analysis		Multivariate Analysis		
	Beta Value	*p* Value	Beta Value	*p* Value	VIF
Age, years	0.168	0.24			
Body mass index, kg/m^2^	−0.557	0.23	−0.461	0.19	1.050
Left ventricular ejection fraction, %	−0.127	0.22			
Mild or greater mitral regurgitation	4.191	0.25			
Serum albumin, g/dL	−3.855	0.15			
eGFR, mL/min/1.73 m^2^	−0.055	0.48			
Plasma B-type natriuretic peptide, pg/mL	0.007	0.007 *	0.004	0.12	1.147
ReDS value, %	1.301	<0.001 *	1.196	<0.001 *	1.098

eGFR, estimated glomerular filtration ratio; ReDS, remote dielectric sensing; VIF, variance inflation factor. * *p* < 0.05 by linear regression analysis. Variables that are considered clinically potential confounders were included in the multivariate analysis after excluding their multicollinearity with VIF < 5.0.

## Data Availability

Data are available upon appropriate request.
